# Silicon Controls Bacterial Wilt Disease in Tomato Plants and Inhibits the Virulence-Related Gene Expression of *Ralstonia solanacearum*

**DOI:** 10.3390/ijms23136965

**Published:** 2022-06-23

**Authors:** Lei Wang, Yang Gao, Nihao Jiang, Jian Yan, Weipeng Lin, Kunzheng Cai

**Affiliations:** 1 Guangdong Provincial Key Laboratory of Eco-Circular Agriculture, Guangzhou 510642, China; kishi218@163.com (L.W.); likoscau@gmail.com (Y.G.); jnhskip@hotmail.com (N.J.); yanjian78@scau.edu.cn (J.Y.); 2 Tea Research Institute, Guangdong Academy of Agricultural Sciences/Guangdong Key Laboratory of Tea Plant Resources Innovation & Utilization, Guangzhou 510640, China; 3 College of Natural Resources and Environment, South China Agricultural University, Guangzhou 510642, China

**Keywords:** silicon, *Ralstonia solanacearum*, tomato, bacterial wilt, biofilm, virulence-related genes

## Abstract

Silicon (Si) has a multifunctional role in improving plant growth and enhancing plant disease resistance, but its mechanisms are not fully understood. In this study, we investigated the impacts of silicon application on the control of bacterial wilt and elucidated the molecular mechanisms using transcriptome sequencing. Compared to non-Si treatment, Si application (0.5–2 mM) significantly reduces tomato bacterial wilt index by 46.31–72.23%. However, Si does not influence the growth of *R. solanacearum*. Si application negatively influences *R. solanacearum* exopolysaccharide (EPS) synthesis and biofilm formation. Transcriptome analysis showed that Si treatment significantly downregulates the expression of virulence genes’ transcriptional regulator (*xpsR*), EPS synthesis-related genes (*epsD* and *tek*), and type III effectors (*HrpB2*, *SpaO*, and *EscR*) in *R. solanacearum*. In addition, Si remarkably upregulates the expression of twitch motor-related genes (*pilE2*, *pilE*, *fimT*, and *PilX*). These findings suggest that silicon-suppressed tomato wilt incidence may be due to the regulation of the virulence-related genes of *R. solanacearum* by Si. Our research adds new knowledge to the application of Si in the field of disease control.

## 1. Introduction

Silicon (Si) accounts for about 28% of the Earth’s crust. Many studies have shown that Si could enhance plant resistance to disease stresses [1,2,3,4]. The mechanisms of Si control plant disease by increasing cell wall strength, activating host defense response through increasing antioxidant enzyme activities or antifungal compounds, and regulating the signal transduction network [5,6,7,8,9]. Si can also directly inhibit the growth of many pathogens, such as *Magnaporthe grisea* [10], *Fusarium solani* [11], *Alternaria solani* [12], and *Fusarium sulphureum* [13] in vitro. Together, Si inhibits pathogen growth by reducing conidia germination and appressorium formation [1,14].

*Ralstonia solanacearum* (*R. solanacearum*) is a soilborne plant pathogen that invades the plant roots from the soil and aggressively colonizes the xylem vessels, which causes wilt symptoms. *R. solanacearum* infection leads to lethal wilting disease in more than 200 plant species and is particularly harmful to Solanaceae plants [15,16]. Many studies report that Si significantly suppresses bacterial wilt in hydroponic, soil, and substrate culture conditions [17,18,19]. However, most of these studies only focus on the interaction between Si and plants, ignoring the effect of silicon on *R. solanacearum*. This pathogen is extremely vigorous and can survive for a long time in soil and water; it can infect different plant tissues through a series of virulence determinants [20]. The virulence factors include plant cell-wall-degrading enzymes, bacterial extracellular polysaccharide (EPS), type III secretion system (T3SS), and motility activity, contributing to the development of wilt disease [21,22]. The expression of virulence factors in *R. solanacearum* is controlled by a complex and precise regulatory network that responds to environmental conditions, host cells, and bacterial density [23]. EPS and T3SS are controlled by a global regulator (*PhcA*). *PhcA* is activated by quorum sensing (QS), and it can induce the expression of *xpsR* and influence the biosynthesis of EPS [24]. 

Some chemical compounds, such as volatile organic compounds and R-methyl 3-hydroxymyristate, can inhibit biofilm formation, EPS production, and *R. solanacearum* colonization in plant roots [25,26]. Tahir et al. (2017) found that *Bacillus* volatiles could reduce bacterial wilt incidence by changing the expression of the virulence factors (*PhcA*, *T3SS*, *ESP*, and chemotaxis) [27]. On the contrary, swimming motility is also a critical factor in bacterial wilt virulence. The virulence of *R. solanacearum* nonmotile mutant (lack of *fliC* or *fliM* gene) was significantly decreased [28]. Yang et al. (2016) found that hydroxycoumarins weakened bacterial wilt virulence by reducing biofilm formation and downregulating flagellar genes such as *fliA* and *flhC* [29]. Several studies have shown that exogenous substances (umbelliferone, hydroxycoumarins, and oleanolic acid) could reduce the infectivity of *R. solanacearum* to host plants by downregulating the expression of *R. solanacearum* virulence factors, which could be applied for the integrated control of bacterial wilt [30,31].

Although some studies have noted that silicon can reduce the infection rate of plants by inhibiting pathogens, the effect of silicon on the virulence of pathogenic bacteria is still unclear. In the present study, our scientific hypothesis is as follows: (1) Si treatment significantly inhibits the expression of *R. solanacearum* virulence genes; (2) Si treatment can reduce *R. solanacearum* EPS synthesis; and (3) Si inhibits biofilm formation of *R. solanacearum*. Therefore, we used transcriptome sequencing to analyze the expression of virulence-related genes of *R. solanacearum* influenced by Si in vitro. In addition, the EPS synthesis and biofilm formation of *R. solanacearum* were analyzed under Si treatment.

## 2. Results

### 2.1. Silicon Application Reduced Disease Index of Bacterial Wilt in Tomato

**Figure 1:**
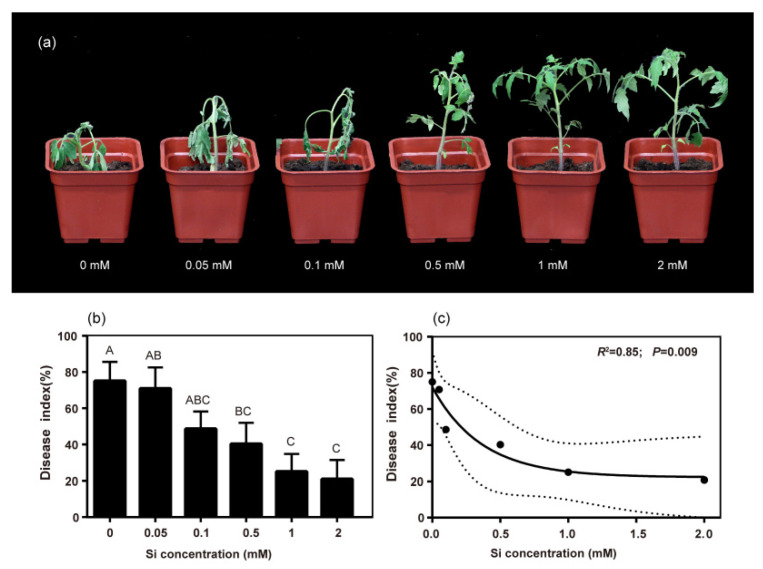
Effects of Si concentration on the disease index of bacterial wilt in tomato. (**a**) Effects of different Si concentrations on tomato plants under *R. solanacearum* inoculation conditions. (**b**) Disease index at 20 days post inoculation (dpi). (**c**) The simple linear regression (solid line) and 95% confidence interval of the regression (dashed line) for the disease index at 20 dpi and Si concentration in peat soil. Different letters among treatments denote statistical difference at *p* < 0.05 according to Duncan’s new multiple range tests.

The low Si concentrations (0.05 and 0.1 mM) did not significantly alleviate bacterial wilt in tomato plants (Figure 1a). However, when the added Si concentration was over 0.5 mM, the disease index of bacterial wilt significantly decreased. Compared to the control (0 mM Si), applying 0.5, 1.0, and 2.0 mM of Si reduced the disease index by 46.31%, 66.67%, and 72.23%, respectively (Figure 1b). Regression analysis showed that the disease index was significantly and negatively correlated with Si concentration (R^2^ = 0.92, *p* < 0.01; Figure 1c).

### 2.2. Silicon Did Not Affect the Growth of R. solanacearum

**Figure 2:**
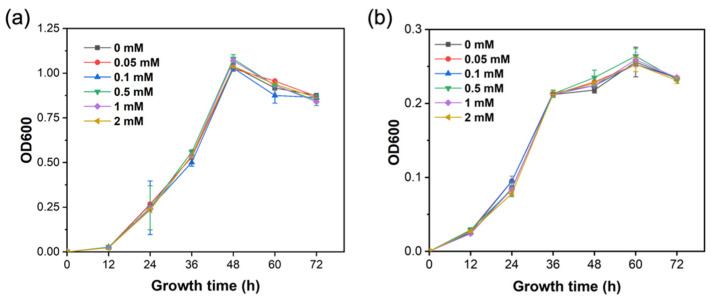
Effects of different Si concentrations on the growth curve of *R. solanacearum*. (**a**) The growth curve of *R. solanacearum* in liquid LB medium. (**b**) The growth curve of *R. solanacearum* in liquid MM medium.

The results show there was no significant difference in the growth of *R. solanacearum* among all treatments (Figure 2). The growth curve of *R. solanacearum* in the liquid Luria–Bertani (LB) and minimal mineral (MM) medium was not significantly influenced by Si treatment with different concentrations, indicating that Si did not have a direct inhibition on *R. solanacearum*.

### 2.3. Silicon Regulated the Expression of 119 Genes of R. solanacearum In Vitro

**Figure 3:**
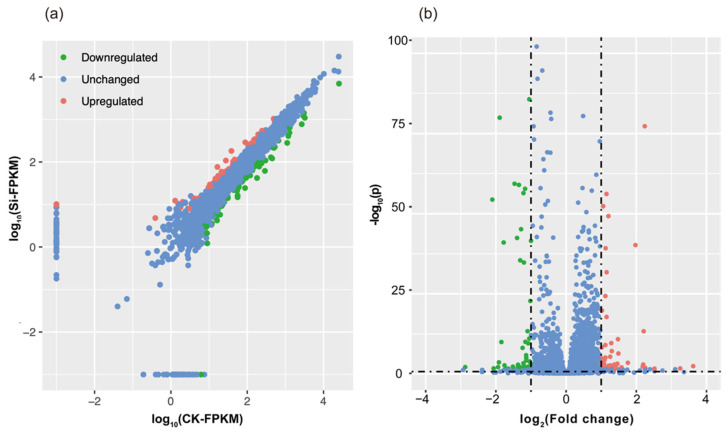
Gene expression changes and associated significant values across different treatments. Scatter plot (**a**) and volcano plot (**b**). Green dots represent significantly downregulated genes {Log_2_FPKM(Si)/FPKM(CK) > 2 and Log_10_FDR [FPMK (Si)/FPKM (CK)]) < 0.05}; red dots represent significantly upregulated genes {Log_2_FPKM(Si)/FPKM(CK) < −2 and Log_10_FDR [FPMK (Si)/FPKM (CK)]) < 0.05}; blue dots represent no DEGs.

The sequencing data were mapped onto the *R. solanacearum* strain GMI1000 reference genome [32], and 4,496 genes were detected (Figure 3). In this study, 119 differentially-expressed genes (DEG) were found to be significantly influenced by Si application, accounting for 2.6% of all known genes. Among these 119 genes, 57 were upregulated, and 62 were downregulated (Tables S1 and S2).

The expression of 15 genes (gene primer was shown in Table S3) related to the virulence of *R. solanacearum* was analyzed by qRT-PCR, including four biological replicates under two housekeeping genes (*GAPDH* and *thyA*). The qRT-PCR data for these genes were significantly correlated with the RNA-Seq results in two housekeeping genes, namely *GAPDH* (Figure S1a) and *thyA* (Figure S1b), which suggested that the RNA-Seq results are reliable. DEGs were compared with the KEGG Orthology database, and the corresponding pathways were established (Table S4). A total of 37 genes (72.5%) in the KEGG pathway database were related to basal metabolism, indicating that the effect of Si on *R. solanacearum* was primarily focused on the basal metabolic process (Figure S2). In addition, 15.68% of the genes were related to the development of environmental information. Si significantly upregulated the expression of the *R. solanacearum* DNA repair gene (*ogt*) and protein repair gene (*degP*, Table S4). Meanwhile, the KEGG BRITE functional annotation showed that 41.81% of genes were involved in enzyme functions, and 23.63% of genes were transporters (Table S5).

### 2.4. Silicon Altered the Transcriptional Expression of xpsR, EPS, and T3SS in R. solanacearum

**Figure 4:**
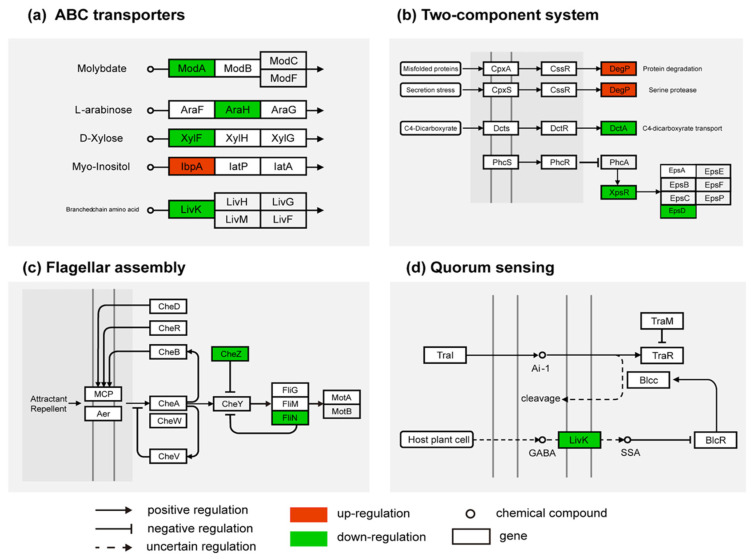
KEGG pathway of DEGs. (**a**) DEGs in the ABC transporter pathway. (**b**) DEGs in the two-component system. (**c**) DEGs in the bacterial chemotaxis pathway. (**d**) DEGs in the quorum sensing pathway. Red: upregulated by Si treatment; Green: downregulated by Si treatment.

DEGs involved in metabolic pathways were analyzed to decipher the molecular mechanism of silicon in influencing *R. solanacearum* virulence. The *ModA*, *AraH*, *XylF*, and *LivK* genes involved in the ABC transporter pathway and EPS synthesis were significantly downregulated by Si (Figure 4a). These genes encoded proteins that mediated the transportation of bacterial molybdate, L-arabinose, D-xylose, and branched-chain amino acid. Moreover, *IbpA*, which was involved in Myo-Inositol transport, was significantly upregulated by Si. In the two-component system pathway, four DEGs (*degP*, *DctA*, *xpsR*, and *epsD*) were enriched (Figure 4b). *degP* was remarkably upregulated by Si treatment. However, *DctA*, *xpsR*, and *epsD* were significantly downregulated by Si application. The pathway analysis results show that the *FliN* gene was related to bacterial chemotaxis and flagellar assembly (Figure 4c).

**Figure 5:**
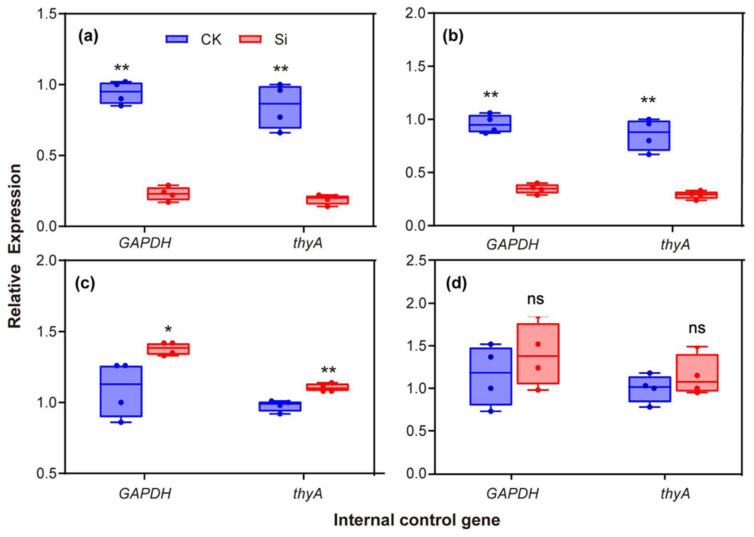
Effects of exogenous silicon on the relative expression of virulence-related genes of *R. solanacearum.* (**a**) *xpsR* (transcriptional regulator). (**b**) *epsD* (the genes coding for EPS). (**c**) *pilE* (the genes coding for twitching motility). (**d**) *fimT* (the genes coding for swimming motility). CK: non-Si treatment; Si: Si treatment. “ns” indicates not significant (*p* > 0.05), * and ** indicate significant difference among treatments at *p* ≤ 0.05 and *p* ≤ 0.01 in *t*-test.

The flagellar assembly pathway is a key pathway downstream of the bacterial chemotaxis. Four genes (*pilE2*, *pilE*, *PilX*, and *fimT*) encoding type-4 fimbriae were downregulated by Si (Table S6). Twelve virulence-related genes were screened, among which four were upregulated, and eight were downregulated (Table S6). Some genes encoding known virulence traits were screened under Si treatment, such as transcription regulator (*xpsR*), EPS-related genes (*epsD* and *tek*), and type III effectors (*HrpB2*, *SpaO*, and *EscR*). The qPCR results show that the expression of the vital virulence genes, namely *xpsR* (2.86 folds) and *epsD* (3.33 folds), were significantly downregulated by Si application (Figure 5a,b). The qPCR results also show that the twitching motility-related gene (*pilE*) was remarkably upregulated by Si treatment (Figure 5c). Furthermore, Si application significantly reduced the expression of *LivK* (Figure 4d).

### 2.5. Silicon Disrupted EPS and Biofilm Formation

**Figure 6:**
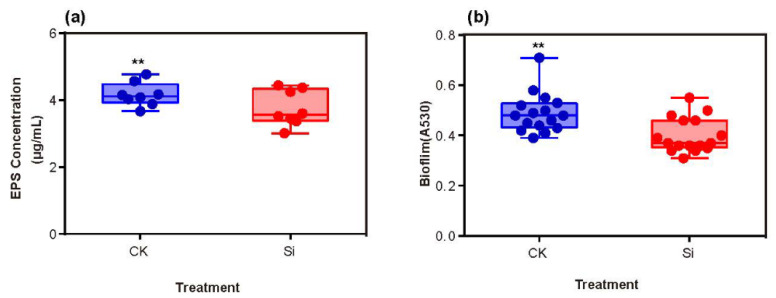
Effects of Si application on EPS concentration and biofilm formation of *R. solanacearum*. EPS concentration (**a**) and biofilm formation (**b**); CK: non-Si treatment; Si: Si treatment. ** indicates significant difference among treatments at *p* ≤ 0.01 in *t*-test.

The *epsD* gene, which was involved in the synthesis of EPS (Table S6), was significantly downregulated by Si application. By contrast, EPS concentration was markedly reduced in 2 mM Si treatment, which was consistent with the transcriptome results (Figure 6a). The biofilm formation was an essential mechanism for bacteria to resist external environmental stress. Our results show that bacterial biofilm synthesis in Si treatment was significantly lower than that of non-Si treatment (Figure 6b).

## 3. Discussion

Multiple studies have investigated the role of Si in controlling bacterial wilt [18,33,34]. Our results show that 0.5–2.0 mM of Si significantly reduced the disease index of bacterial wilt by 46.31%–72.23% (Figure 1a,b), which is consistent with previous studies. However, our study found that Si did not significantly inhibit the growth of *R. solanacearum* (Figure 2a,b). Previous studies demonstrated that exogenous additives (such as benzimidazole and vitamin C) could prevent pathogens from infecting the host not by directly suppressing pathogens, but by reducing the bacterial virulence, which weakens the ability of bacteria to infect the host [35,36].

Our study showed that Si could significantly downregulate EPS synthesis-related genes, namely, *epsD* and *tek* (Table S6, Figure 5b). EPS is also a key virulence factor of *R. solanacearum* and part of the exopolysaccharide operon, which is the primary substance used by *R. solanacearum* to block the xylem of plants, and the mutants of the EPS gene lose their virulence ability [37,38]. Minic et al. (2007) reported that *epsD* affected EPS biosynthesis in *Streptococcus thermophilus*, and *epsD* mutant did not produce EPS [39]. *tek* is an extracellular protein associated with *EPS1* and regulated by *PhcA* [40]. 

Our studies showed that EPS production decreased in Si treatment (Figure 6a). Therefore, we suggest that Si inhibited the expression of *espD* and *tek*, thereby reducing *R. solanacearum* EPS synthesis. Notably, other genes, except for *epsD* in the *eps* gene cluster of *R. solanacearum*, are not significantly expressed in our experiment. Different genes in the same gene cluster have a different substrate specificity, so there will be differences in the expression levels in the same environmental conditions. In order to uncover the exact reason why silicon inhibits EPS synthesis of *R. solanacearum*, more molecular experiments need to be carried out in future research.

Biofilms are microbial defense and communication systems [41]. The results show that the formation of *R. solanacearum* biofilm was significantly inhibited by Si treatment (Figure 6b). Kong et al. (2018) demonstrated that a benzimidazole derivative (UM-C162) prevented the formation of *Staphylococcus aureus* biofilm, but this derivative had no effect on bacterial viability, and the transcriptome analysis showed that UM-C162 treatment inhibited the expression of bacterial biofilm formation and bacterial attachment-associated genes [42]. Hence, Si inhibited *R. solanacearum* biofilm synthesis, which might weaken bacterial virulence.

In our study, the transcriptome results show that Si treatment significantly downregulated the expression of eight genes (*HrpB2*, *SpaO*, *EscR*, *xpsR*, *tek*, *epsD*, RSc2755, and RSp1004), which were primarily related to bacterial virulence regulation (Table S6). *HrpB2*, *SpaO*, and *EscR* were type III effectors’ proteins. T3SS were used to inject effectors proteins into plant cells and delivered collections of type III effectors proteins to weaken host defenses [43]. The *HrpB2* gene belongs to the *hrp* gene cluster, and it is an essential component of T3SS [44]. The expression of *HrpB2*, *SpaO*, and *EscR* genes were downregulated by Si treatment, indicating that T3SS of *R. solanacearum* was inhibited, which may lead to a reduction in bacterial virulence. The *xpsR* gene is controlled by *PhcA*, which regulates EPS production [45]. 

Considerable evidence suggested that the *LysR*-type regulatory factor *PhcA* gene was the core of the virulence regulatory network of *R. solanacearum* [33,46,47]. *PhcA* is a *LysR*-type transcriptional regulator, which can regulate virulence factors, activate EPSs and cellulase, and inhibit the mobility of *R. solanacearum* [48]. Chen et al. (2015) found that the expression of *R. solanacearum* virulence-related genes (*xpsR*, *tek*, and *epsE*) was significantly inhibited in the *PhcA* mutant. Collectively, Si suppressed the expression of virulence-related genes, which might decrease the infection rate of *R. solanacearum* in tomato [45].

Twitching motility is an important bacterial behavior that allows pathogens to efficiently migrate and colonize host plants. Our study also found that the type-4 fimbriae genes, *pilE2*, *pilE*, *PilX*, and *fimT*, were significantly upregulated by Si (Table S6). The swimming motility of *R. solanacearum* can be affected by flagella, which are related to bacterial virulence [49]. The flagellar motor switch is composed of three proteins: *FliG*, *FliM*, and *FliN*, which can control flagellum’s rotation [50]. Our study found that Si inhibited the expression of *CheZ* and *FliN* (Figure 4c), indicating that Si enhanced the movement of fueling flagella and promoted the twitching motility of bacteria. Twitching motility is important to pathogens in avoiding stressful environments.

## 4. Materials and Methods

### 4.1. Experimental Materials

The *R. solanacearum* strains (GMI1000) and tomato seeds (genotype HYT, bacterial wilt-susceptible tomato) were provided by Professor Guoping Wang (College of Horticulture, South China Agricultural University, Guangzhou, China). Si was applied in the form of anhydrous potassium silicate (K_2_SiO_3_, Thermo Fisher Scientific, mean weight ratio: SiO_2_:K_2_O = 2.5).

### 4.2. Effects of Different Si Concentrations on Wilt Incidence

Tomato seeds were surface sterilized with 10% H_2_O_2_ for 10 min, followed by rinsing three times with ultrapure water. Sterilized seeds were germinated in petri dishes containing two layers of filter paper for 48 h at 30 °C. The germinated tomato seeds were transferred to plug trays containing sterilized peat soil (Klasmann, Germany), grown in an incubator (30 °C, RH 80%, 12 day/12 night, MGC-400B, Yiheng-Shanghai, China), and watered daily with 1/2 concentration tomato special nutrient solution (each liter of nutrient solution contains 5 mM Ca(NO_3_)_2_, 1.88 mM K_2_SO_4_, 1.63 mM MgSO_4_, 0.5 mM KH_2_PO_4_, 0.04 mM H_3_BO_3_, 0.001 mM ZnSO_4_, 0.001 mM CuSO_4_, 0.01 mM MnSO_4_, 0.00025 mM Na_2_MoO_4_, 0.05 mM NaCl and 0.1 mM Fe-EDTA). 

Tomato seedlings at the third-leaf stage were transplanted into the pot (5 cm × 5 cm × 8 cm) containing 1 kg of sterilized peat soil. There are six treatments, including the 0, 0.05, 0.1, 0.5, 1.0, and 2.0 mM Si, with twelve replications in this experiment. Tomato seedlings were irrigated daily with 50 mL of 1/2 concentration tomato special nutrient solution (containing 0, 0.05, 0.1, 0.5, 1.0, and 2.0 mM Si) in each pot. The non-silicon treatment was supplemented with a corresponding amount of KCl to balance the increased K element in the Si nutrient solution.

Tomato plants at the sixth-leaf stage were inoculated with pathogens. *R. solanacearum* strains were grown on B medium (Difco Peptone 10 g·L^−1^, yeast extract 1 g·L^−1^, and casamino acids 1 g·L^−1^) at 28 °C and 150 r·min^−1^ [51]. After 48 h, bacterial cells were collected and centrifuged three times with sterilized water. *R. solanacearum* suspension was resuspended in deionized water and adjusted to OD600 = 0.1 (10^8^ cfu·mL^−1^) with a spectrophotometer (Shimadzu UV-2600, Kyoto, Japan). Then, 50 mL of the bacterial suspension was poured into each pot. The disease index of tomato plants was recorded from the wilting of the first leaf and continuously recorded for 20 d.

### 4.3. Effects of Different Silicon Concentrations on the Growth of R. solanacearum

The growth curves of *R. solanacearum* in LB and MM medium were investigated, according to a modification of the method described by Lowe-Power et al. (2018) [52]. There were six treatments: 0, 0.05, 0.1, 0.5, 1.0, and 2.0 mM Si. *R. solanacearum* strains were grown on liquid LB medium at 28 °C and 150 r·min^−1^ for 24 h. The cultured cells were adjusted to OD600 = 0.1 using sterile water, and 200 μL of bacterial suspension was added to 30 mL of liquid LB and MM medium. The medium was incubated at 28 °C with shaking at 150 rpm for 72 h in an incubator-shaker (HZQ-X300, Yiheng-Shanghai, China). Growth rate was assessed by measuring OD600 values every 12 h. All experiments have four replicates.

### 4.4. Pathogen Symptom Evaluation

The disease index was determined according to the methods described by Chen et al. (2015) [33]. A disease index was recorded from the first wilting leaf of tomato plants. Grade 0: asymptomatic; Grade 1: 1 leaf half wilting; Grade 3: 2 to 3 leaves wilting; Grade 5: all leaves are wilted except for the top 1 to 2 leaves; Grade 7: all leaves are wilting; Grade 9: the leaves and plants die.

### 4.5. EPS Assay

EPS was extracted from *R. solanacearum* strain [53]. *R. solanacearum* strain was grown at 30 °C for 48 h in LB medium. Five milliliters of overnight cultures of *R. solanacearum* were adjusted to OD600 = 0.1, then *R. solanacearum* was added to 95 mL of sterile liquid MM medium (with and without Si medium) and cultured with shaking for 48 h at 28 °C. *R. solanacearum* cell suspension (10^8^ CFU·mL^−1^) was centrifuged at 12,000 rpm for 10 min. The supernatant was filtered by a 0.22 μL filter membrane. The filtrate was placed in a lyophilizer overnight to freeze dry, then 1 mL of 95% ethanol was added, and the mixture was kept at 4 °C for 24 h. Then, the mixture was centrifuged at 5000 r·min^−1^ for 10 min to collect the precipitate, and 1 mL of water was heated to dissolve the residue and obtain a crude EPS solution. The purified EPS was diluted to 25 mL with distilled water and frozen to store for later use. EPS was determined using the Elson–Morgan method [54].

### 4.6. Biofilm Assay

The biofilms of *R. solanacearum* were measured in vitro with a minor modification of the polyvinyl chloride microtiter plate assay [55]. Briefly, *R. solanacearum* strain was grown at 30 °C for 48 h in LB medium. Then, 5 mL of *R. solanacearum* suspension (OD600 = 1.0) was added to 45 mL of MM liquid culture broth (with and without Si treatment). Two hundred microliters of culture solution were added to 96-well polystyrene microtiter plates. After the 96-well plates with culture solution were incubated at 28 °C for 48 h, the culture medium was carefully removed, and the biofilm was washed twice with 200 μL ultrapure water. The 96-well plates with bacterial membrane were dried at 60 °C for 30 min to fix the bacterial membrane. Then, 200 μL of 0.1% crystal violet was added to stain the biofilm for 30 min, and the culture solution was washed two times with 200 μL of distilled water to move the crystal violet. Then, 95% ethanol was used to adsorb the crystal violet from the biofilm. The solution was measured for absorbance at 530 nm.

### 4.7. Transcriptome Analysis

*R. solanacearum* strain was grown at 30 °C for 48 h in LB medium. Five milliliters of *R. solanacearum* suspension were adjusted to OD600 = 0.1 and were added into an Erlenmeyer flask containing 50 mL of MM medium with or without 2 mM of Si. Potassium chloride was used to adjust potassium differences between controls and treatments. There were six samples for each treatment. The medium was incubated at 28 °C with shaking at 150 rpm for 48 h, and bacterial cells were collected for transcriptome analysis.

RNA was extracted using the Bacterial RNA Kit (Tiangen Biotechnology, Beijing, China) according to the manufacturer’s recommendations. Preliminary quantification and accurate quantification were detected using a NanoDrop 2000 spectrophotometer (Thermo Fisher Scientific, Inc., Wilmington, DE, USA) and electrophoresis concentration Agilent 2100 RNA 6000 Nano Kit (Figure S3), respectively. Twenty microliters of RNA per treatment were taken for cDNA library construction. The construction of cDNA libraries and RNA-Seq was performed by Genedenovo Bio-Tech Co., Ltd. (Guangzhou, China).

### 4.8. Data Quality Check and Analysis

The high-throughput sequencing and preliminary analysis of the data were completed by Guangzhou Gideo Biotechnology Co., Ltd. The raw data obtained from Illumina HiSeqTM 2500 sequencing were converted into sequenced reads by CASAVA base calling and stored in FASTQ file format. Raw sequences with adaptors and unknown nucleotides above 5% or those that were of low quality were removed to obtain clean reads. The filtered reads of ribosomes were compared with the reference genome by TopHat2 version 2.0.14 [56]. Cufflinks version 2.2.1 [57] was used to assemble transcripts based on reference annotation-based transcripts. 

Fragments per kilobase million (FPKM) value estimation was used to measure gene expression. RSEM version 1.3.3 [58] was used to count the bowtie’s comparison results. The DEGseq version 1.18.0 [57] was applied to normalize the FPKM values among samples for statistical analysis. DEGs were screened based on the criteria of ∣log2 Ratio∣ ≥ 1 and Q ≤ 0.05. *p*-value was determined by controlling the false discovery rate. DEGs were also submitted to the GO program (http://www.blast2go.org (accessed on 22 June 2022)) for functional annotation [59]. We used KEGG (http://www.kegg.jp/ (accessed on 22 June 2022) pathway analysis to compare and enrich differential genes and determine the main biochemical metabolic pathways and signal transduction processes.

### 4.9. qRT-PCR

qRT-PCR was performed to validate the RNA-Seq results for 15 gene transcripts. The qPCR experiment used the SYBR Premix Ex Taq Kit (Takara, Japan), and qPCR was measured by the ABI Step One Plus Real-Time PCR System (Applied Biosystems, Foster City, CA, USA). The reaction system (total volume, 20 μL) included 10 μL of SYBR Premix Ex Taq II, 0.6 μL of PCR Forward Primer (10 μM), 0.6 μL of PCR Reversed Primer (10 μM), 2.0 μL of cDNA, and 2.8 μL of ddH_2_O. The amplification cycling program was as follows: 90 s at 95 °C, followed by 40 cycles at 95 °C for 5 s, 60 °C for 15 s, and 72 °C for 20 s. Relative quantification was used for the conversion of gene expression. Data were analyzed using the 2^−^^ΔΔCt^ method [60]. Two internal reference genes (*GAPDH* and *thyA*) were selected, and each sample was subjected to four technical replicates. The primers used for qPCR are listed in Table S3.

### 4.10. Statistical Analysis

All data were statistically analyzed by *t*-test and Duncan’s method for multiple comparisons at a 5% significance level. All the statistical analyses were performed with SPSS 20.0 (IBM, Chicago, IL, USA).

## 5. Conclusions

In this study, we found that Si significantly suppressed bacterial wilt not by directly inhibiting the growth of *R. solanacearum*, but by influencing the expression of virulence-related genes. Si also reduced the synthesis of *R. solanacearum* EPS and biofilm. On the contrary, Si upregulated *R. solanacearum* flagellar genes that promoted bacterial twitching motility. Hence, we hypothesized that Si had a negative influence on *R. solanacearum*. In the presence of Si treatment, Si downregulated virulence-related genes (*HrpB2*, *SpaO*, *EscR*, *xpsR*, *tek*, and *epsD*) of *R. solanacearum* and inhibited *R. solanacearum* EPS synthesis. Meanwhile, Si promoted *R. solanacearum* movement-related genes (*pilE2*, *pilE*, *PilX*, and *fimT*). However, the specific mechanism by which silicon inhibits the virulence factors of *R. solanacearum* remains unclear. Further research needs to be conducted with *R. solanacearum* mutants or plant infection experiments. This study provides a new perspective to decipher the role of silicon in controlling plant diseases.

## Data Availability

The data presented in this study are available on request from the corresponding author.

## Supplementary Materials

The following supporting information can be downloaded at: https://www.mdpi.com/article/10.3390/ijms23136965/s1.
