# Supplementary material for: Silicon Controls Bacterial Wilt Disease in Tomato Plants and Inhibits the Virulence-Related Gene Expression of Ralstonia solanacearum
Source: Int J Mol Sci. 2022 Jun 23;23(13):6965. doi: 10.3390/ijms23136965 (PMC9266643; doi:10.3390/ijms23136965)
Supplement: Supplementary file 1 [file ijms-23-06965-s001.zip › ijms-1719652-supplementary.pdf]

Silicon Controls Bacterial Wilt Disease in Tomato Plants and Inhibits the Virulence-Related Gene Expression of *Ralstonia solanacearum*

Lei Wang [1], Yang Gao [1], Nihao Jiang [1], Jian Yan [1], Weipeng Lin [2,*] and Kunzheng Cai [1,3,*]

[1] Guangdong Provincial Key Laboratory of Eco-Circular Agriculture, Guangzhou 510642, China; kishi218@163.com (L.W.); likoscau@gmail.com (Y.G.); jnhskip@hotmail.com (N.J.); yanjian78@scau.edu.cn (J.Y.)

[2] Tea Research Institute, Guangdong Academy of Agricultural Sciences/Guangdong Key Laboratory of Tea Plant Resources Innovation & Utilization, Guangzhou 510640, China

[3] College of Natural Resources and Environment, South China Agricultural University, Guangzhou 510642, China

* Correspondence: linweipeng@gdaas.cn (W.L.); kzcai@scau.edu.cn (K.C.); Tel.: +86-20-88523253 (W.L.); +86-20-38297175 (K.C.)

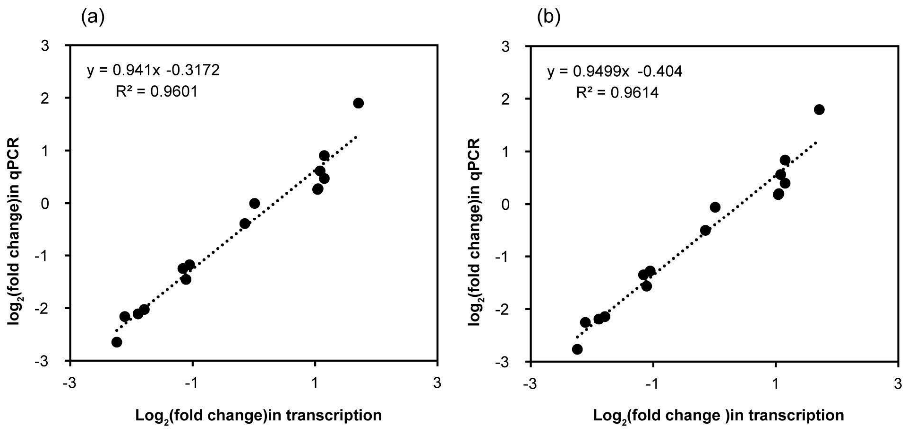

Figure S1. Correlation of transcription (x axis) and qPCR data (y axis) for a subset of 15 DEGs. (**a**) *GAPDH* gene as internal control. (**b**) *thyA* gene as internal control.

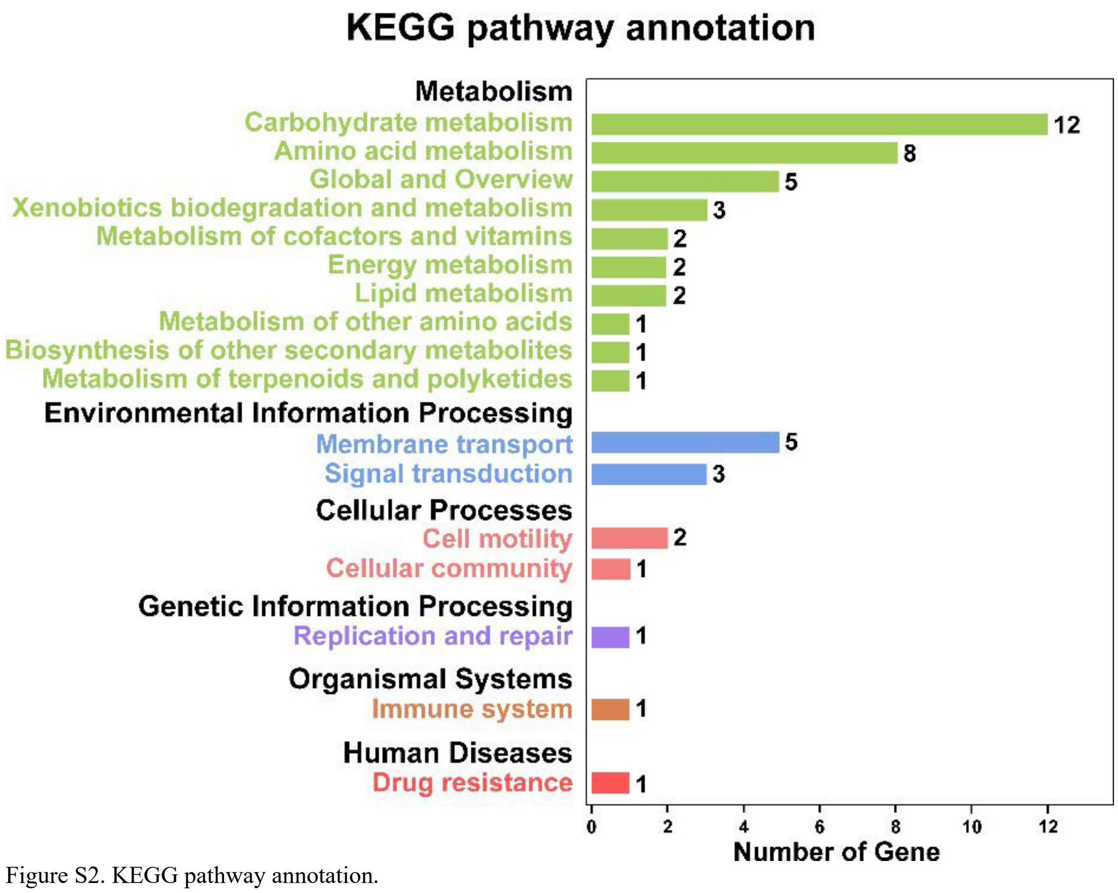

Figure S2. KEGG pathway annotation.

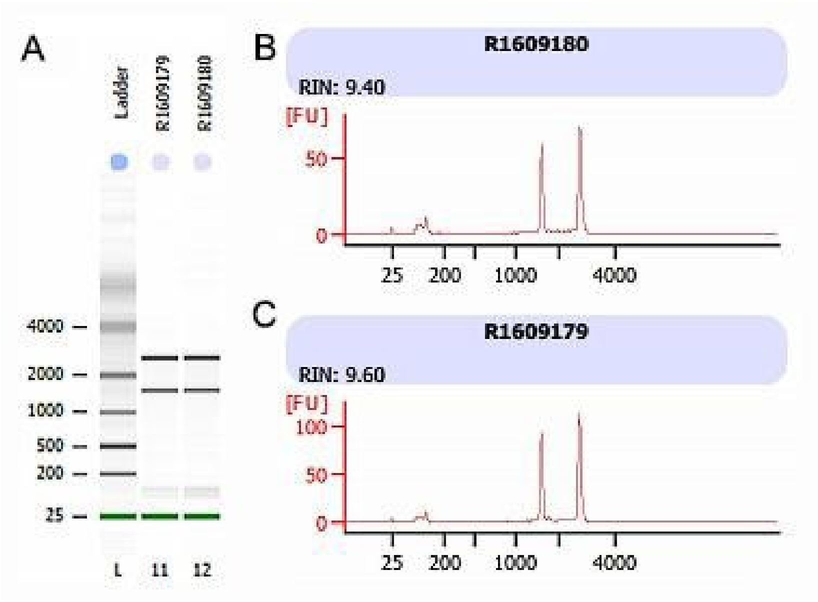

Figure S3. Quality of the extracted RNA. A: Capillary electrophoresis of the samples; B: Integrity of CK samples; C: Integrity of Si samples.

Table S1 The genes significantly upregulated in Si treatment compared with CK

No.	Gene ID	log2(FC)	*p*-value	No.	Gene ID	log2(FC)	*p*-value
1	RSc0699	13.32	1.56E-02	30	RSc2519	1.21	1.67E-02
2	RSp1151	13.27	1.56E-02	31	RSc0199	1.2	5.42E-03
3	RSc3187	13.05	7.82E-03	32	RSc1963	1.2	7.78E-50
4	RSc1895	3.62	1.84E-03	33	RSc0583	1.2	6.67E-03
5	RSc3357	3.25	1.17E-02	34	RSc1325	1.19	1.38E-02
6	RSp0632	2.52	1.30E-02	35	RSp0002	1.15	4.54E-19
7	RSc1850	2.26	7.56E-03	36	RSc2080	1.15	1.02E-32
8	RSp1108	2.24	5.13E-77	37	RSc1057	1.15	1.76E-56
9	RSp1107	2.21	1.08E-14	38	RSc0609	1.13	4.75E-40
10	RSc1241	2.2	5.51E-04	39	RSp1106	1.12	1.87E-25
11	RSp0379	2.18	2.61E-03	40	RSc0593	1.12	2.96E-06
12	RSp0564	1.97	5.41E-41	41	RSc1254	1.12	2.65E-10
13	RSp1464	1.76	1.45E-04	42	RSp0602	1.09	4.13E-03
14	RSp1552	1.71	3.79E-11	43	RSc0723	1.09	1.88E-05
15	RSp0199	1.58	4.58E-03	44	RSc2740	1.09	1.21E-02
16	RSp1638	1.49	1.37E-07	45	RSc1614	1.08	3.53E-04
17	RSc0076	1.48	4.08E-12	46	RSc0374	1.08	1.56E-18
18	RSc1025	1.46	2.94E-04	47	RSc2780	1.06	6.08E-06
19	RSc2388	1.45	4.68E-04	48	RSp1449	1.06	8.09E-53
20	RSc1622	1.41	9.10E-03	49	RSc2680	1.05	1.46E-05
21	RSc1159	1.38	5.33E-04	50	RSc0722	1.04	2.25E-04
22	RSp0757	1.36	1.62E-02	51	RSc0719	1.03	1.55E-03
23	RSc0512	1.33	1.28E-03	52	RSc2528	1.02	4.68E-21
24	RSp0443	1.3	2.30E-08	53	RSc2880	1.01	1.49E-09
25	RSp0708	1.3	3.84E-03	54	RSc0570	1.01	4.37E-04
26	RSc3150	1.29	6.87E-11	55	RSc2725	1.01	3.44E-04
27	RSc2280	1.26	1.59E-06	56	RSc2629	1	3.61E-05
28	RSc2506	1.26	1.11E-03	57	RSc0980	1	4.71E-06
29	RSp0713	1.21	1.32E-03				

Table S2 The genes significantly downregulated in Si treatment compared with CK

No.	Gene ID	log2(FC)	*p*-value	No.	Gene ID	log2(FC)	*p*-value
1	RSp0937	-12.58	3.91E-03	32	RSc0093	-1.26	0.00E+00
2	RSc0626	-2.88	4.20E-03	33	RSc1093	-1.26	8.01E-03
3	RSc3301	-2.24	0.00E+00	34	RSp0479	-1.24	2.01E-09
4	RSp0106	-2.1	8.82E-55	35	RSp1001	-1.22	2.35E-92
5	RSp1373	-2.07	1.19E-02	36	RSp1005	-1.21	9.87E-07
6	RSc2984	-1.99	1.13E-16	37	RSc2313	-1.21	1.94E-04
7	RSp1510	-1.94	3.74E-03	38	RSp0306	-1.2	1.59E-03
8	RSc2641	-1.91	1.00E-04	39	RSc2981	-1.2	1.06E-35
9	RSc2983	-1.89	1.25E-79	40	RSp0836	-1.17	4.34E-08
10	RSp1002	-1.87	0.00E+00	41	RSc2756	-1.16	2.47E-11
11	RSp0982	-1.85	3.02E-11	42	RSc3232	-1.16	1.34E-02
12	RSp1003	-1.79	0.00E+00	43	RSp1249	-1.14	4.90E-07
13	RSp0319	-1.78	8.93E-42	44	RSp0837	-1.13	3.61E-06
14	RSp0482	-1.75	1.08E-03	45	RSp0838	-1.13	2.37E-05
15	RSp0377	-1.73	1.47E-02	46	RSp0829	-1.11	1.28E-02
16	RSc0190	-1.73	1.47E-02	47	RSp1016	-1.11	4.10E-15
17	RSp1526	-1.71	2.05E-18	48	RSp0140	-1.11	6.53E-03
18	RSc0931	-1.65	1.26E-02	49	RSc2980	-1.1	1.09E-14
19	RSp1004	-1.56	0.00E+00	50	RSc2113	-1.1	3.11E-03
20	RSp1268	-1.55	1.74E-21	51	RSc2755	-1.08	7.61E-11
21	RSp1366	-1.52	2.92E-03	52	RSp0971	-1.07	1.77E-02
22	RSc2982	-1.47	1.26E-59	53	RSp0650	-1.07	5.22E-03
23	RSp0484	-1.4	3.64E-43	54	RSc0538	-1.05	2.55E-12
24	RSp1000	-1.39	0.00E+00	55	RSp1633	-1.05	4.03E-85
25	RSc0597	-1.37	5.95E-04	56	RSp0485	-1.04	5.42E-05
26	RSp0696	-1.37	2.22E-03	57	RSc1825	-1.03	8.74E-03
27	RSc1750	-1.36	9.54E-03	58	RSp0649	-1.02	1.28E-02
28	RSp1527	-1.35	2.97E-59	59	RSp1636	-1.02	5.85E-24
29	RSp1401	-1.33	3.22E-03	60	RSp0995	-1.01	2.55E-04
30	RSc3128	-1.31	2.37E-36	61	RSp1011	-1.01	3.95E-23
31	RSp0483	-1.28	7.65E-46	62	RSp1530	-1	2.86E-42

Table S3 Gene primer sequence for qPCR

Accession	Gene		Primer Sequence（5'-3'）
RSc2749	*GAPDH*	F	CGCCATCGCCAACTTCAA
		R	GTTGACGACCAGCGTATTGC
RSc0947	*thyA*	F	GGCGACTGCCATCTCTACAAC
		R	GCGGGATGGCTTTCGTAG
RSc2553	*argG*	F	CCTATGCCTACACCGCCAA
		R	CGTGGTGTTGAAGTAGGTGATG
RSc0374	*rpoH*	F	GCCGAGATGCACGAGTACA
		R	CCACCGCATCGACTTCGT
RSc0722	*fimT*	F	AAGCAGCCGCAGGTCACT
		R	GACAAGGAGGTGAGGCAGG
RSc1057	*rseB*	F	GGTGGCATTCTCGCAAGTC
		R	AGCCCGTCACGCTCTTCA
RSc2680	*pilE*	F	CGGTTCTGGCGTATCCCT
		R	TCGTGAGGTCCGAGGCAT
RSc2748	*phcA*	F	CCTACAACCTCAACGCCTTCC
		R	CAGCATCGGGTGCGTGTT
RSc2756	*araH*	F	TGATCGCCCGGCTGAAG
		R	TGGTGTTGCCCAGGTCG
RSc2983	*rpoE*	F	GCGTGGCTGTTGACCATCA
		R	TCGGTAGGCCAGTTGCTCC
RSc3301	*putA*	F	CGCCATGACGGAAGAGGA
		R	GCTTGATCGAGATGCCCG
RSp0002	*cspD3*	F	AAAGGCTTCGGCTTCATCA
		R	CTTGATACCCGAGGCTTGC
RSp0106	*modA*	F	GACCAATGCCTTCAAGACGC
		R	GCCTTGTCCATCGCTTCCT
RSp1003	*xpsR*	F	CGGTGGCAATCGCATCA
		R	CTTCCAGTCGCATTCGCTCT
RSp1016	*epsD*	F	ATCGCCTTCGCCAATGAG
		R	GGCTTGGCATCGTTGACC
RSp1552	*degP*	F	ACCTCCATCCAGGGTTTGC
		R	ATTGCTTGTCCACCCCCA
RSp1633	*xylF*	F	GGGCATCAAGGTGGTCTCG
		R	GGCGTTGTTGTCGGTGGA

Table S4 DEGs function definition in KEGG database

No.	Gene ID	KEGG ID	Gene Name	Function Definition	Patten
1	RSc0093	K01251	*ahcY*	adenosylhomocysteinase	down
2	RSc0190	K01420	*fnr*	CRP/FNR family transcriptional regulator, anaerobic regulatory protein	down
3	RSc0626	K07302	*iorA*	isoquinoline 1-oxidoreductase subunit alpha	down
4	RSc1093	K08195	*pcaK*	4-hydroxybenzoate transporter	down
5	RSc1750	K01999	*livK*	branched-chain amino acid transport system substrate-binding protein	down
6	RSc1825	K00450		gentisate 1,2-dioxygenase	down
7	RSc2755	K13874	*araB*	L-arabinonolactonase	down
8	RSc2756	K10538	*araH*	L-arabinose transport system permease protein	down
9	RSc2980	K17247	*msrQ*	methionine sulfoxide reductase heme-binding subunit	down
10	RSc2981	K07147	*msrP*	methionine sulfoxide reductase catalytic subunit	down
11	RSc2983	K03088	*rpoE*	RNA polymerase sigma-70 factor, ECF subfamily	down
12	RSc3128	K00138	*aldB*	aldehyde dehydrogenase	down
13	RSc3301	K13821	*putA*	RHH-type transcriptional regulator, proline utilization regulon repressor	down
14	RSp0106	K02020	*modA*	molybdate transport system substrate-binding protein	down
15	RSp0319	K01534	*zntA*	Cd-exporting ATPase	down
16	RSp0377	K02417	*fliNY*	flagellar motor switch protein FliN/FliY	down
17	RSp0479	K02028		polar amino acid transport system ATP-binding protein	down
18	RSp0483	K01750	*ocd*	ornithine cyclodeaminase	down
19	RSp0485	K01091	*gph*	phosphoglycolate phosphatase	down
20	RSp0649	K00020	*mmsB*	3-hydroxyisobutyrate dehydrogenase	down
21	RSp0650	K00140	*mmsA*	methylmalonate-semialdehyde dehydrogenase	down
22	RSp0696	K04127	*cefD*	isopenicillin-N epimerase	down
23	RSp0829	K01706	*gudD*	glucarate dehydratase	down

24	RSp0836	K03462		nicotinamide phosphoribosyltransferase	down
25	RSp0937	K02160		acetyl-CoA carboxylase biotin carboxyl carrier protein	down
26	RSp0995	K11103	*dctA*	aerobic C4-dicarboxylate transport protein	down
27	RSp1003	K19667	*xpsR*	transcriptional activator of eps genes	down
28	RSp1016	K02472	*epsD*	UDP-N-acetyl-D-mannosaminuro nic acid dehydrogenase	down
29	RSp1366	K03734	*apbE*	FMN transferase	down
30	RSp1373	K19342	*NosL*	copper chaperone	down
31	RSp1401	K03414	*cheZ*	chemotaxis protein	down
32	RSp1633	K10543	*xylF*	D-xylose transport system substrate-binding protein	down
33	RSc0199	K12262	*cybB*	cytochrome b561	up
34	RSc0374	K03089	*rpoH*	RNA polymerase sigma-32 factor	up
35	RSc0570	K05786	*rarD*	chloramphenicol-sensitive protein	up
36	RSc0583	K03753	*mobB*	molybdopterin-guanine dinucleotide biosynthesis protein	up
37	RSc0722	K08084	*fimT*	type IV fimbrial biogenesis protein	up
38	RSc0980	K07240	*chrA*	chromate transporter	up
39	RSc1025	K04755	*fdx*	ferredoxin, 2Fe-2S	up
40	RSc1057	K03598	*rseB*	sigma-E factor negative regulatory protein	up
41	RSc1241	K17213	*IbpA*	inositol transport system substrate-binding protein	up
42	RSc1963	K03832	*tonB*	periplasmic protein TonB	up
43	RSc2506	K00567	*ogt*	methylated-DNA-[protein]-cystei ne S-methyltransferase	up
44	RSc2528	K03561	*exbB*	biopolymer transport protein	up
45	RSc2680	K02655	*pilE*	type IV pilus assembly protein PilE	up
46	RSc2780	K03181	*ubiC*	chorismate--pyruvate lyase	up
47	RSp0002	K03704	*cspA*	cold shock protein	up
48	RSp0379	K02415	*fliL*	flagellar FliL protein	up
49	Rsp1151	K01821		4-oxalocrotonate tautomerase	up
50	RSp1464	K02003		putative ABC transport system ATP-binding protein	up
51	RSp1552	K04771	*degP*	serine protease Do	up

Table S5. DEGs in KEGG BRITE database annotation

No.	KOID	Function Orthology	GeneNo.
1	ko01000	Enzymes	23
2	ko02000	Transporters	13
3	ko02035	Bacterial motility proteins	4
4	ko03000	Transcription factors	4
5	ko03021	Transcription machinery	3
6	ko02044	Secretion system	3
7	ko03400	DNA repair and recombination proteins	1
8	ko04147	Exosome	1
9	ko03110	Chaperones and folding catalysts	1
10	ko01002	Peptidases	1
11	ko01009	Protein phosphatases	1

Table S6. DEGs in virulence genes of *R. solanacearum*.

No.	Gene ID	Gene name	Pattern	Pathogenesis
1	RSp1016	*EpsD*	down	EPS synthesized key genes
2	RSp1003	*xpsR*	down	Transcription regulator protein
3	RSp0140	*HrpB2*	down	Type III effector
4	RSp0838	*SpaO*	down	Type III effector
5	RSp0837	*EscR*	down	Type III effector
6	RSp1002	*tek*	down	EPS transmembrane transport
7	RSc2755		down	EPS synthesis-related genes
8	RSp1004		down	EPS synthesis-related genes
9	RSc2680	*pilE2*	up	type-4 fimbrial biogenesis transmembrane protein
10	RSc0719	*PilX*	up	type-4 fimbrial biogenesis pilx-related signal peptide
11	RSc0722	*FimT*	up	type-4 fimbrial pilin related transmembrane protein
12	RSc0723	*PilE*	up	type-4 fimbrial pilin-related transmembrane protein
